# Process evaluation of a co-design and implementation study to improve professional health literacy in a regional care hospital (PIKoG): a mixed-methods study

**DOI:** 10.1186/s12913-025-12679-9

**Published:** 2025-04-15

**Authors:** Johanna Sophie Lubasch, Hannah Nordmann, Mona Voigt-Barbarowicz, Sonia Lippke, Christina Derksen, Anna Levke Brütt, Lena Ansmann

**Affiliations:** 1 Department of Health Services Research and Research Network Emergency and Intensive Care Medicine, School of Medicine and Health Sciences, Carl von Ossietzky University Oldenburg, Ammerlaender Heerstrasse 140, Oldenburg, 26129 Germany; 2 Department of Health Services Research, School of Medicine and Health Sciences, Carl von Ossietzky University Oldenburg, Oldenburg, Germany; 3 Hamburg University of Applied Sciences/ Hochschule für Angewandte Wissenschaften Hamburg (HAW Hamburg), Hamburg, Germany; 4 School of Business, Social & Decision Sciences, Constructor University Bremen gGmbH, Bremen, Germany; 5 Wolfson Institute of Population Health, Queen Mary University of London, London, UK; 6 Department of Medical Psychology, University Medical Center Hamburg-Eppendorf, Hamburg, Germany; 7 Chair of Medical Sociology, Institute of Medical Sociology, Health Services Research and Rehabilitation Science (IMVR), Faculty of Medicine, University of Cologne, Cologne, Germany

**Keywords:** Communication training, Complex intervention, MRC framework, Organizational development, Organizational health literacy

## Abstract

**Background:**

In connection with a hospital stay, patients have to make important health-related decisions. Adequately responding to the needs of patients requires good communication skills of healthcare professionals within healthcare organizations. The PIKoG project (As made for us – Improving professional health literacy in hospitals) aimed at improving professional health literacy by implementing participatory health literacy training and supporting measures in a hospital setting. This study aimed to analyze processes supporting and hindering the implementation of the complex intervention.

**Methods:**

A mixed-methods study was conducted, including focus group interviews and a paper-pencil survey with healthcare professionals. Data was combined and analyzed using categories derived from the Medical Research Council’s guidance on process evaluation: (1) Implementation, (2) Mechanisms of impact, and (3) Context. Interview data were analyzed using structured qualitative content analysis according to Kuckartz. Survey data were analyzed descriptively.

**Results:**

One of three on-site, full-day health literacy training sessions was offered weekly. Supporting measures were implemented step by step over the course of a year. Both the training and the supporting measures were rated positively overall, but they could not be effectively integrated into daily routines. The COVID-19 pandemic as well as resource constraints adversely affected implementation by altering workflows, increasing stress levels and shifting priorities. The participatory approach and individual change agents fostered the implementation of the complex intervention. Nurses were reached the most, while physicians engaged least in the interventions. Adaptations during the implementation increased the use of the implemented measures and gave rise to ideas for future improvements.

**Conclusion:**

The study highlights the challenges involved in implementing a complex intervention supporting professional health literacy in an organization and stresses the importance of considering available resources, recruiting opinion leaders, and being responsive to the needs of different groups. While the participatory co-design development approach was found to be valuable, it does not guarantee successful organizational change in times when hospitals face multiple challenges. Subsequent studies should therefore focus on investigating the capacities of healthcare organizations for organization-wide improvement processes and identify how healthcare organizations can be innovative and patient-centered even in the presence of extremely difficult contextual conditions.

**Trial registration:**

DRKS00019830, since 16th of April 2020.

**Supplementary Information:**

The online version contains supplementary material available at 10.1186/s12913-025-12679-9.

## Background

There has been increasing recognition of the importance of health literacy to improve patients’ health outcomes [1]. Patients must be able to find, understand, assess and apply health-related information. This is especially important when making health-related decisions [2, 3]. Low health literacy is associated with increased hospitalization, lower adherence to medication use, poorer general health and higher mortality in geriatric patients [4]. Research has shown that more than 55% of the German population have problematic or insufficient health literacy, with similar results being found for other European countries [5–7]. While it is known that health literacy is based on personal competencies and correlates with socio-demographic factors, in recent years, more attention has been drawn to the complexity of the healthcare context [6, 8, 9]. The increasing complexity of the healthcare system is especially challenging to navigate for patients with low health literacy [2]. Hospitalization poses a particular challenge for patients, as they have to give up parts of their autonomy, privacy and their familiar surroundings when they are admitted to hospital and at the same time have to make important health-related decisions [10, 11]. To facilitate access to health information and orientation, healthcare organizations such as hospitals must therefore integrate principles of health literacy-sensitive care in both infrastructure and communication processes; this is captured by the concept of Health-Literate Healthcare Organizations [8]. Part of this concept is professional health literacy, i.e., the knowledge and skills that healthcare professionals need to (i) acquire and manage professional knowledge, (ii) prepare, explain and communicate health-related information in ways that patients can understand, appraise and apply, (iii) engage patients and help them participate in decision making, and (iv) support them in the use of digital health information [12]. Evidence from a sample of German hospitals indicates that organizational health literacy is not yet embedded in inpatient care. There is especially a need for improvement in terms of health literacy trainings for healthcare professionals [13–15]. A review of Kaper et al. [16] revealed that interventions have the potential to lead to positive changes on health literacy and as a result on patient outcomes. A health-literate hospital for example makes it more likely to enhance the patient-provider relationship, to address patients’ unmet information needs during their hospital stay and to improve domains of individual health literacy [16, 17]. However, the implementation of these interventions is challenging due to conflicting or low priorities and awareness of the topic, lack of resources, high complexity of the implemented intervention elements, or lack of change agents as key persons driving the change within the organizations [16, 18, 19].

The PIKoG project (As made for us – Improving professional health literacy in hospitals [German: Wie für uns gemacht - **P**artizipativ angelegte **I**mplementierung eines **Ko**mmunikationskonzepts zur Verbesserung der professionellen **G**esundheitskompetenz]) aimed to improve professional health literacy with an organization-wide complex intervention to thereby foster organizational health literacy. The study was conducted in acute inpatient care at a community hospital in northwestern Germany by implementing a communication and health literacy concept for healthcare professionals as well as supporting measures for patients and healthcare professionals from October 2019 to January 2023 (see Appendix 1 and 2 for intervention components). The intervention was defined as complex according to the Medical Research Council (MRC) framework [20] as it was composed of various adjustable components that required a high number of actions by the implementation team and those receiving the intervention. Since healthcare settings are complex organizations [21], basic principles of organizational development – such the adaption of the complex intervention to the specific organization and the involvement of organizational members in the change process in a codesigning manner – were considered in intervention development and implementation [21, 22]. More precisely, communication and health literacy training sessions developed across Europe [23] were adapted in a co-creative mode by means of needs assessment, stakeholder workshops and pilots [24]. Since change agents have been found to be important drivers of change within organizations [16, 18, 19], a group of key healthcare professionals in the organization was established as health literacy change agents (HLCAs) to increase implementation success [25].

The presented implementation and the process evaluation are based on the MRC framework for conducting and reporting process evaluations of complex intervention studies [26]. The framework builds on three themes of process evaluation: (1) Implementation, (2) Mechanisms of impact, and (3) Context. The themes thereby are related to each other, for example, the implementation of the new intervention is affected by the context in which it is implemented and change in turn impacts of the context in which it is delivered. The framework suggests when measuring implementation to subdivide it into the four aspects, namely reach (whether the intended audience comes into contact with the intervention, and how), dose (the quantity of intervention implemented), fidelity (whether the intervention was delivered as intended), and necessary adaptions to fit the intervention [20]. Mechanisms of impact is defined by how the delivered intervention produces change. Context is defined by how the setting affects implementation and outcomes [26]. Regarding the planning phase, stakeholders should be involved and the design and causal assumptions of the interventions should be (1) Implementation, (2) Mechanisms of impact, and (3) Context [26]. The framework recommends to use a combination of qualitative and quantitative data for the process evaluation [26].

While co-designed interventions within healthcare organizations are more likely to be used and sustained [21, 27, 28], complex interventions aiming to improve patient care still often fail [29]. This is due to implementation hurdles and lack of consideration of contextual factors such as staffing, resources or climate [29]. To investigate these factors and improve future implementation, a process evaluation is needed. Process evaluations are an integral part of the updated MRC framework for developing and evaluating complex interventions [20], including process evaluation [26]. Process evaluation focuses on answering questions of quality and fidelity of implementation. It explores how change is achieved and helps understand why an intervention fails or succeeds by analyzing the context in which the intervention is implemented. By analyzing the implementation process and examining unexpected pathways, process evaluations can facilitate future development and implementation of the intervention in another context [20]. The evaluation thereby aims to detect potential challenges as well as opportunities that were faced during the implementation process. The findings can serve future studies implementing complex interventions in the field of organizational health literacy.

The aim of this study was therefore to evaluate the barriers and facilitators, including contextual factors, regarding the implementation of a complex intervention promoting professional health literacy in a hospital setting. The following questions were investigated:

1. How was the complex intervention implemented from the point of view of the healthcare professionals?
2. Which barriers and facilitators played a role during the implementation process of the complex intervention?
3. How do the healthcare professionals evaluate individual components of the complex intervention?

## Methods

### Description of the intervention and implementation

The PIKoG study consisted of three (partly parallel) phases: 1. Development phase, 2. Implementation phase and 3. Evaluation phase. In the development phase, a communication and health literacy concept was designed using a participatory approach. In the implementation phase, this concept was implemented in the hospital setting. In the evaluation phase, effectiveness as well as the implementation process were examined in order to identify mechanisms of action and contextual factors. A detailed description of the phases can be found in the previously published study protocol [30].

Different stakeholders and influential healthcare professionals from different professions within the hospital and clinical departments were identified as HLCAs in the development phase of the project. The role the change agents played in the project was twofold. On the one hand, the HLCAs should contribute to the planning of the intervention by bringing in the perspective of the hospital staff; on the other hand, the HLCAs should promote the implementation of the intervention in the hospital and build a bridge between the research team and the staff. Therefore, they participated in meetings to raise awareness and interest in the study, discussed roles and responsibilities, and developed a cooperation agreement. To ensure the intervention program’s success, steering board meetings were conducted during the implementation phase with these partners to create a sense of ownership and to coordinate tasks throughout the study.

The intervention itself entailed a communication and health literacy concept with two parts. Firstly, a co-designed communication and health literacy training program was implemented for healthcare professionals [23] (see Appendix 1). The training was based on previously evaluated training units that were effective in improving healthcare professionals’ knowledge about health literacy [23, 31]. Adapted to current needs, three full-day training sessions were developed: (1) “Communication Skills for Patient Interaction”, (2) “Patient-Centered Communication” and (3) “Team Communication” [24] which all focused on the link between communication and health literacy. The three full-day on-site communication training sessions were offered alternately on a weekly basis over a period of twelve months. The trainings sessions were designed independently from each other, but all employees of the participating clinics were asked to participate in all three trainings. To participate in the training, the employees were given paid leave of absence from work. Secondly, supporting measures were developed (see Table 1) and implemented in the hospital environment to improve the conditions for promoting health literacy. Examples of the supporting measures can be found in Appendix 2. The supporting measures were supposed to be targeting everyone on the wards.

**Table 1:** Co-designed supporting measures

Supporting measure	Description	Aim	Location	
Poster (A3 Format)	Displays communication techniques covered in training [24]	Deepen knowledge of techniques	Posted on the wards, e.g., physicians’ lounge, ward room, conference room	
Index cards (A7 Format)	Display communication techniques learned in training [24] in brief	Remind of techniques	Fit in coat pockets of work clothes	
Door signs	“Do not disturb” signs for multiple contexts	Prevent disturbances during rounds or confidential conversations	Available at the nurses’ station	
Communication cards (tip doc by Setzer)	Sheets using a combination of pictures and short sentences in multiple languages	Facilitate communication with patients who are not proficient in German, without spoken language or with reading/writing difficulties	Available at the nurses’ station	
Communication portfolio (UKAPO)^*^	Pictograms of the body and medications	Facilitate diagnosis and communication about the correct use of medication	Available at the nurses’ station	
Flyer for patients	Flyer on how to find good health information on the internet	Increase the level of patient information	Available at the waiting areas for patients	
Flyer for patients	Flyer motivating patients to note questions prior to consultations	Prepare patients for their conversation with the healthcare professionals	Available at the waiting areas for patients	
Explanation videos	Explain diseases and operations	Provide easy-to-understand patient information	Published on the hospital’s website	

^*^UKAPO = symbol cards supporting augmentative and alternative communication in pharmacies (German = unterstütze Kommunikation in der Apotheke)

**Fig. 1:**
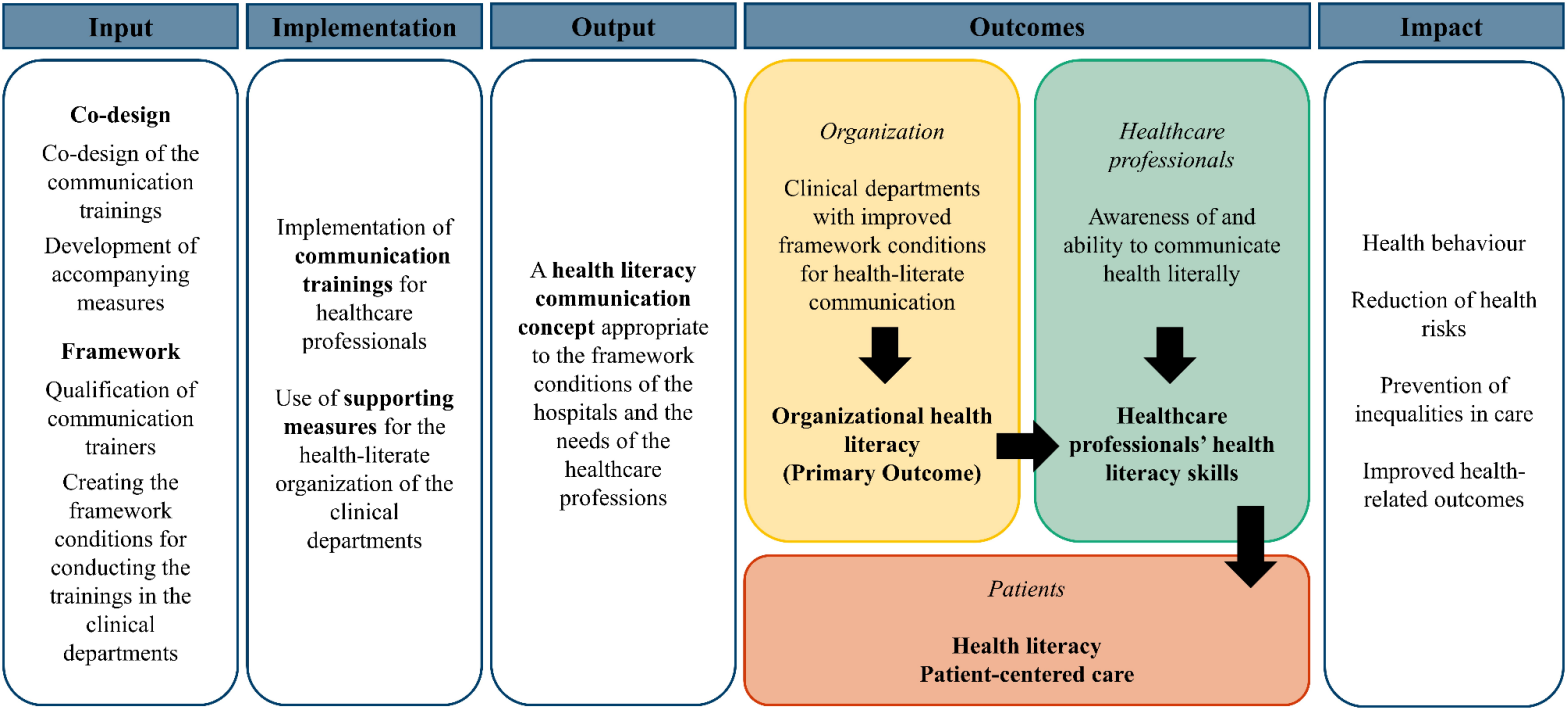
Logic model of the PIKoG intervention [21, 30]

The logic model (see Fig. 1) has been specified in the study protocol and shows how the intervention was thought to produce change by improving professional health literacy [30]. The development of the intervention was based on the MRC framework. As recommended, a logic model was set up to clarify the intervention itself and its causal assumptions [26]. Details on the logic model can be found in the study protocol previously published by Lubasch et al. [30].

### Study design

The PIKoG intervention was delivered and tested in a mixed-methods intervention study, conducted in acute inpatient care at a community hospital in northwestern Germany. For the process evaluation, a convergent parallel mixed-methods design was chosen by collecting qualitative and quantitative data and interpreting the results of the two sets in a merged form. The participating hospital is part of the University Medical Centre Oldenburg. Compared to most university hospitals, the participating hospital with 420 beds and selected care areas is not a maximum-care provider, but can be described as a regional care hospital located in a medium-sized city (approx. 170.000 inhabitants) in a rural region. Moreover, not all clinical departments of the hospital are part of the University Medical Centre. Therefore, only four clinical departments were involved in the study that were part of the University Medical Centre when the study was planned: Orthopedics and Trauma Surgery; Gynecology; Internal Medicine – Oncology; Visceral Surgery. The participating hospital was merged with another hospital during the study.

In this study, the implementation was being systematically evaluated by looking at the following parts of the MRC framework by Moore et al. [26]: context, fidelity, reach/dose, necessary adaptations, and mechanisms of impact. *Context* refers to all external circumstances that affect the intervention and how it is implemented. *Fidelity* describes whether the implementation of the intervention is delivered as intended. *Reach/dose* refers to the quantity of the intervention implemented and if intended persons are reached by it. *Necessary adaptions* describe whether the intervention itself or the implementation needs any changes to improve the intervention. *Mechanism of impact* refers to how change is achieved. Data was collected within a mixed-methods approach at the level of health professionals at the end of the implementation phase.

### Data collection

#### Sample size calculation/planning

About 20 individuals were aimed to include in the qualitative data collection: Qualitative data was collected from multiple professions to enable contrasting perspectives and was used to inform the development of the quantitative survey.

Additional 120 employees were aimed to include in the quantitative data collection. These numbers were based on and communicated in our protocol paper and trial registration [30].

#### Qualitative data

Qualitative data was collected during semi-structured focus group interviews (see Appendix 3). The aim of the focus groups was to identify what barriers and facilitators emerged during the implementation of the complex intervention as well as to determine reach, fidelity, mechanisms of impacts, and the need for necessary adaptations of the intervention components. The MRC framework served as basis to develop the guide of the interviews [26] (see Appendix 3). Participants were recruited through leaders of the clinical departments, the research team, research assistants from the participating departments, and involved HLCAs. Health professionals were interviewed if they gave written consent. Aligned with the method of purposive sampling, it was targeted to recruit the participants heterogeneously regarding their profession and work experience. The focus group interviews were conducted for each professional group separately to ensure that the participation in and openness of the discussion is not influenced negatively by professional hierarchy. Due to the current developments in the Covid-19 pandemic at the time, the interviews were conducted online. To enable discussion about all intervention components, the participants received pictures of and information on the components beforehand via mail.

#### Quantitative data

**Fig. 2:**
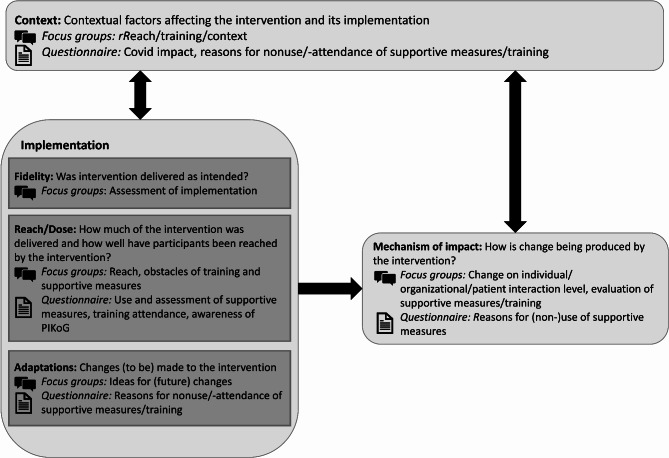
Integration model based on Moore et al. [20, 26]

Quantitative data was collected to assess the frequency of use and assessment of the supporting measures, training attendance, awareness of the PIKoG project as well as reasons for nonuse of supportive measures and nonattendance of the training sessions (see Fig. 2). Printed surveys were handed out to leaders of all four participating clinical departments as well as social workers and patient information staff, physiotherapists, and psycho-oncological staff. Participation was voluntary, and participants provided written informed consent. During team meetings, the health professionals were reminded to participate in the survey. Two weeks after the survey started, the research team increased recruitment efforts by providing information signs as well as sweets with the project logo printed on them in the ward rooms as incentives. The survey collected information about reach, dose in terms of use of supporting measures and training [24], information about barriers in using the supporting measures as well as a short assessment of each supporting measure (see Table 2 for sample items). To measure participants’ readiness to change, the shortened German version of the Change Attitude Scale, validated by Hower et al. was used [32]. The nine-item scale has a Cronbach’s alpha of 0.79. Values range from 0 (negative change attitude) to 3 (positive change attitude). Due to the specific nature of the intervention, all further questions regarding the process evaluation in the questionnaire were self-developed. To ensure differentiated collection of information, items included whether intervention components were recognized (yes/no), how often the components were approximately used if suitable (e.g. never, once month, once a week, 2–3 times a week, every day) and if it was used, how helpful the component was (5-point Likert scale, from not at all to fully).

**Table 2:** Survey items of the questionnaire

	Number of questions/items	Origin of questions/items	Example item	Answer options
Reach	1	Self-developed	How aware have you been of the PIKoG project at the hospital for you personally?	5-point scale (not at all to fully)
Dose	5^*^	Self-developed	How often did you use the index cards on communication techniques of the PIKoG project?	6-point scale (once a week to not at all)
Barriers	5^*^	Self-developed	Why did you not use the door signs?	Multiple answer options (e.g. did not know about them, no time, no interest) and one open text field
Assessment	5^*^	Self-developed	How helpful were the communication cards in your daily clinical routine?	5-point scale (not at all to completely)
Readiness to Change	9	Hower et al. [32]	I was open to consider and try out the change	4-point scale (fully disagree to fully agree)

^*^One for each intervention component developed to be used by the healthcare professionals

### Data analysis

#### Qualitative data analysis

All focus group interviews were transcribed verbatim and imported into MAXQDA Analytics Pro 2022 [33] for data analysis. Structured qualitative content analysis according to Kuckartz [34] was used to analyze the data to break down the complexity of the material and identify categories. Accordingly, in all focus groups, main categories were first developed deductively á priori on the basis of the interview guide. Since the guide was based on the MRC framework, the deductive categories were: reach, dose, fidelity and adaptions, context, and mechanism of impact. Afterwards, inductive subcategories were developed in the coding process with information from the transcribed interview material itself [34]. This procedure was first tested by two researchers based on two interviews. The inductively formed subcategories were compared, and the category system was developed. Subsequently, the two researchers coded all interviews independently and compared the results in a consensus-finding process.

#### Quantitative data analysis

A descriptive quantitative data analysis was conducted using IBM SPSS for Macintosh version 27.0 [35]. Participants who answered less than 50% of all mandatory questions were removed from the dataset because it was assumed that data is to incomplete to be included into the survey. Since this was only one person, no imputations were conducted.

#### Integration

During the interpretation phase, quantitative and qualitative data were analyzed together to explore the five dimensions adapted from the MRC framework. The quantitative data allowed us to assess the utilization of the supporting measures and communication and health literacy training [24] as well as the impact of the COVID- 19 pandemic on working conditions. The qualitative data provided additional information about the five dimensions, especially on the context and mechanisms of impact. As reach and dose are closely related and both are being measured by training attendance as well as use of supporting measures, they were combined for the interpretation. While adaptation usually refers to changes that must be made when implementing in a different context, healthcare professionals’ suggestions for creating sustainable change at their hospital were also included in this category. Figure 2 gives an overview of the key dimensions and how they were measured in this study, inspired by Moore et al. [26].

## Results

### Sample characteristics

In total, 12 healthcare professionals were interviewed in four focus groups: Group 1 - Physicians (*n* = 2), Group 2 – Nursing staff (*n* = 4), Group 3 – Social workers and physiotherapists (*n* = 2) and Group 4 – Hospital management and administration staff (e.g., quality management, marketing) (*n* = 3).

The highest participation rate in the training was among nursing staff (47.4%), followed by the “others” group (26.3%) which mainly consisted of support staff (e.g., patient navigators). Of 221 eligible healthcare professionals, 77 completed the survey (34.8% response); out of these, 76 (98.7%) answered at least 50% of all mandatory questions and were included in the analysis. Among these respondents, 32.9% were under 29 years old, while 39.5% were over 50 years old, 78.9% were female and 47.4% were nurses (see Table 3). Participants tended to be ready for changes in the organization with a mean change attitude of 2.58 with a *SD* of 0.3, on a range from 0 to 3.

**Table 3:** Sample characteristics of the healthcare professional survey

	*n* = 76	%	Mean (SD)
***Gender***			
Female	60	78.9	
Male	15	19.7	
Other	1	1.3	
***Profession***			
Nursing staff	36	47.4	
Physician	16	21.1	
Therapist	4	5.3	
Others	20	26.3	
***Department and Experience***			
Visceral surgery	34	44.7	
Orthopedics and Trauma Surgery	19	25.0	
Gynecology	18	23.7	
Outpatient	11	14.5	
Internal Medicine – Oncology	2	2.6	
Years of work experience			17.8 (13.3)
Years working at this hospital			12.0 (11.6)

### Dimensions adapted from the MRC framework

#### Context

From the interviewees’ perspective, the COVID-19 pandemic was the most important contextual factor affecting the delivery of the training sessions in 2021. Due to safety concerns, many employees, especially physicians, were hesitant to participate in in-person training. Fear of infection was frequently cited as a reason for not attending these events.

*“For me*,* fear was part of the reason*,* I wanted to be with people as little as possible” – Physician*

The COVID-19 pandemic was also experienced to have a profound impact on workflows and schedules, making it difficult for some healthcare professionals to attend the training. Nearly 70% of healthcare professionals interviewed agreed that the COVID-19 pandemic had significantly changed workflows and tasks. According to interviewees, this was especially true for small teams or those with part-time employees, where lack of time was a major barrier to participation. The time available for training was experienced to be limited according to staff, and sending staff to these training sessions could have resulted in disruption of patient care, especially during times of staff shortage.

*“We tried to work around the days and the work hours*,* but it was hard because of the staff situation. The motivation of nurses was also not at 100%; you had to really encourage them.” – Nurse*

This is also reflected in the survey, where 52.7% of the respondents cited lack of time or staffing as a reason for not attending training, and 71% agreed that the COVID-19 pandemic had significantly impacted their work lives.

#### Fidelity

Regarding the implementation of the complex intervention, it became clear in the focus groups that the HLCAs had difficulties in getting support from their colleagues. One of the biggest problems was the lack of support from the leadership of the university departments who had initiated the project.

*“The university clinics launched a project. But I don’t think they understood the scope and what it actually means for departments to participate*,* what resources are needed. I don’t think they thought it through to the end” *– Hospital Management and Administration

From the interviewees’ perspective, this was reflected by the fact that interviewees did not feel encouraged by the leadership to participate in the training sessions, which did not relieve them from their routine duties. The interviewees further felt that they had not sufficiently internalized the intervention content to inspire others to change. They noted that despite the training and supporting measures, employee behavior did not change and described a significant amount of effort to implement the methods learned in the training in everyday work routines. As planned, the supporting measures were repeatedly presented in the training, but the participants in the focus groups experienced them as getting lost in daily work. Two factors contributed to this problem. Firstly, it was reported that employees who did not participate in the training were unfamiliar with the materials. Secondly, even among those who did attend the training, a lack of routine led to insufficient use of the supporting measures:

*“We received the communication cards to communicate with the patients. Yes*,* they are lying nicely in a drawer.” – Nurse*

The training sessions were continuously evaluated and adapted according to the needs of the participants to ensure a codesign process. This was appreciated by the employees and supported the implementation from their point of view.

#### Reach and dose

According to the survey, 28.9% of respondents indicated that the PIKoG project was moderately present in the hospital, while 31.6% felt it was fairly or completely present. However, 18.4% of respondents indicated that they were not aware of the project at all. The interviewees mentioned that the contact with the research during the development and implementation team was positive, as this ensured greater visibility of the project.

According to the interviewees, the group of physicians was mainly involved in the project during the planning phase but did not participate in the training, which was criticized by the nursing staff:

“*I have not once seen an XYZ [anonymized] in any training session. Not once. None of them knew what PIKoG even is. And that’s such a shame*,* especially since it was announced by the attending physician at the time*,* but nothing happened.*” – Physician

Overall, the changes achieved were estimated to be minimal and limited to the individual level:

*“Whether anything has changed overall in the hospital? I don’t think so. Well*,* just too few people have participated so far for that. So*,* for me at least*,* it’s not that I would say*,* oh yes*,* since*,* I think*,* since just about a year ago*,* there has been a noticeable improvement in communication with patients on the wards. I wouldn’t say that.” – Physiotherapist*

The participation rate in the training was low overall and much lower than planned (Table 4). Several reasons were provided for this. In the focus groups, a small team and lack of time were frequently cited as reasons. Of 39 training sessions that were planned, 14 (35.9%) had to be cancelled at short notice because the number of participants was too small, or the registration period was too short. It was also experienced that those who most needed the training did not attend, as one social worker explained:

**Table 4:** Training participation

Training	Participants^a^	Participants from clinical departments of PIKoG project^b^
1. Communication Skills for Patient Interaction	34	16
2. Patient-centered Communication	38	21
3. Team Communication	44	18

^a^Of about 150 employees

^b^Training sessions were open to all employees of the hospital

*“Those who find it difficult to engage with patients don’t come either (laughs).” – Social worker*

Among training attendees, nurses were the most widely represented occupational group, while physicians were least represented. The ratio of participating nurses to physicians from the clinical departments of the PIKoG project was 8:1, whereas the ratio of nurses to physicians employed in the departments is approximately 3:1.

*“From nursing*,* many people were there*,* social workers*, etc.,* were also represented*,* in part the psycho-oncologists*,* physiotherapists. So*,* we had the other professional groups that work at the bedside*,* but mostly nurses were there and very*,* very few medical professionals*,* to bring up the topic of interprofessional or multidisciplinary communication again.” – Hospital Management and Administration*

The reach and dose of the supporting measures varied depending on the measure. When asked how often the various supporting measures were used in daily clinical practice, the most common response for all measures was “never.” Door signs were the most likely to be used, with 19.7% of respondents using them more than once a week (Table 5). A total of 64.5% looked at posters on communication techniques which were posted on the wards.

**Table 5:** Use of supporting measures

“How often did you use …”	Index cards	Door signs	Communication cards & portfolio
*N* = 76	*n (%)*	*n (%)*	*n (%)*
Never	60 (78.9)	47 (61.8)	54 (72)
Fewer than 1x per month	12 (15.8)	7 (9.2)	14 (18.7)
1x per month	3 (3.9)	2 (2.6)	3 (4)
2 - 3x per month	0 (0)	2 (2.6)	3 (4)
1x per week	0 (0)	3 (3.9)	0 (0)
More often than 1x a week	1 (1.3)	15 (19.7)	1 (1.3)

The use of the supporting measures varied between the clinical departments.

#### Adaptations

During the study, the training units were continuously adapted to ensure codesign based on participant feedback. The research team also received continuous feedback on the supporting measures. For example, while the door signs were widely used, the physiotherapists found them too large. As a result, smaller signs were designed to fit in the pockets of their work clothes to accommodate their concerns.

*“I was shocked when I got the stack of signs. And the size was crucial*,* you can’t take them with you!” – Physiotherapist*

Several suggestions emerged from the focus groups on how to increase effectiveness and to make PIKoG sustainable. The nurses expressed a desire for supervision and a communication expert to accompany patient conversations and provide feedback on their communication behavior. Alternatively, they suggested creating opportunities for joint reflection, e.g., discussing communication problems during shift handovers. Overall, the desire was repeatedly expressed to implement more reminders during the workday to encourage reflection on communication and health literacy.

*“So*,* a big wish for me would be if you were allowed to accompany us*,* also regarding data protection*,* to accompany conversations and then have a reflection for the respective teams: “How do you talk to each other*,* how do you talk to the patients*,* how is an issue to be managed in communication?” – Nurse*

To address the problem of low attendance at training sessions, participants suggested several solutions, including shorter training sessions to accommodate part-time workers, holding training sessions at off-site locations with coffee and snacks, and offering more frequent sessions over a longer period of time. Some suggested tailoring the training to the specific needs of different occupations or offering online options to avoid COVID-19 pandemic risks.

*“Making it a little bit fancier*,* having to go somewhere. The rooms here*,* they are okay*,* but I think they have to get out of the house. No phones should ring and so on. It has to almost be private*,* meaning civilian clothes and then leave.” – Hospital management and administration*

However, some participants also appreciated the personal aspect of the training. Participants also suggested making the training less complex and focusing on topics with which health professionals are already familiar to deepen their knowledge. In addition, some suggested making the training mandatory to ensure that all staff attend and reflect on their behavior, even those who do not personally identify a need for improvement. The various suggestions were discussed with the HLCAs during the implementation process, and it was decided to shorten the training sessions to make them more attractive.

#### Mechanisms of impact

All in all, the communication and health literacy concept was evaluated to be good, however, the participants experienced the implementation to have failed.

*“I think it’s more a problem of implementation. So*,* and the acceptance within the hospital*,* the realization. And not so much the theory behind it*,* and also the concept*,* which I think is very*,* very good. It’s more that we haven’t accepted it yet*,* because as employees we haven’t seen the need for it.” – Physician*

**Fig. 3:**
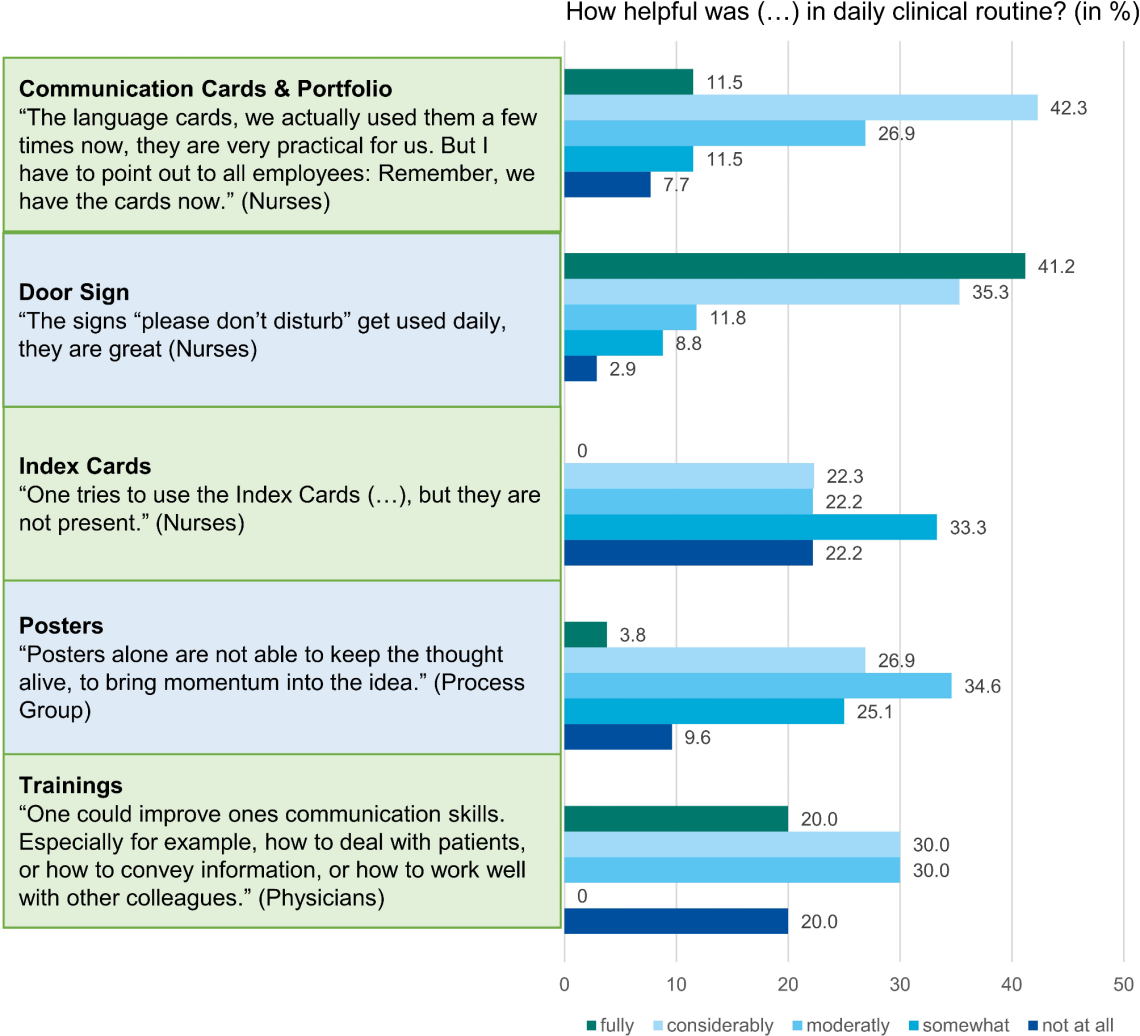
Helpfulness of communication training and supporting measures: results from focus groups and the survey

Also, the individual intervention components were rated positively by the interview partners. The supporting measures were rated as helpful by employees who had participated in the training in reflecting on their behavior and skills. For example, the very simple measure of the door signs has had a very positive effect (also see Fig. 3).

*“So*,* I notice that when I come out of the patient room and someone is standing there and asks: “Can I come in now?“. So*,* they definitely have an effect and conversations can take place more freely than before. So*,* I find the signs helpful.” – Social worker*

Also, the communication cards were rated to be helpful, when they were used (also see Fig. 3).

*“The cards*,* I can say again now*,* we’ve actually used them a few times now*,* the ones with the different languages*,* they’re very practical for us*,* because we use them a lot.” – Nurse*

The training sessions were also rated very positively overall and had the particular effect of raising awareness of the issue of communication with patients and within the team and strengthening the participants’ competencies (also see Fig. 3).

*Those who took part in the training really came back with positive reinforcement. They really multiplied that*,* and then the others said*,* “Oh*,* then I’ll go there too.” – Nurse*

The third training session, “Team Communication,” allowed employees to reflect on their team culture and communication.

*“So*,* I think that a little more attention is paid to the climate (…) so that the rounds can be carried out undisturbed and someone doesn’t come through the door every two seconds. We’ve had this problem before” – Physician*

Although the interviewees experienced the training to have raised awareness of patients’ communication needs and contributed to better team communication, they did not observe any clear effects at the overall hospital level.

In the interviews, one physician indicated that they did not see a need for communication and health literacy training. The reasons they gave were their many years of experience in communicating with patients and the teaching of communication skills in medical school.

*“I also had the impression that someone would now come in and explain to the chef how to boil water*,* because that is our everyday life.” – Physician*

Moreover, the focus group data showed that the implementation of the supporting measures was experienced as insufficient to bring about a perceptible change.

*“Well*,* when one remembered them*,* and used them*,* they were good supporting measures” – Nurse*

The participants faced the challenge of transferring what they had learned into their daily work and remembering the intervention components (see Fig. 3).

*“It’s not the supporting measures themselves*,* it’s not the places they are stored*,* it’s us. We have to spread and raise awareness*,* create opportunities to use them. To integrate them into day-to-day life and to live it.” – Nurse*

## Discussion

The aim of this study was to evaluate the barriers and facilitators, including contextual factors, regarding the implementation of a complex intervention promoting professional health literacy in a hospital setting. The process evaluation guided by the model developed by Moore et al. [26] revealed multiple factors that influenced the implementation of communication and health literacy training sessions [24] and supporting measures. While the overall evaluation of the complex intervention and the attitude towards change were generally positive, the sustainability of this change was lacking.

As the main contextual barrier, the COVID-19 pandemic changed the workflow in the hospital. For example, in-person training became difficult. While an organization can never be expected to be stable when implementing complex interventions, the pandemic posed an exceptional challenge. During the implementation process, only crucial medical and healthcare staff, but not research staff, had access to the hospital, thus potentially limiting the visibility of the project. Training participation was affected by the pandemic, e.g., due to the minimization of social contacts. One possible option would have been to transfer the training into an online format [36]. However, this option was rejected by the HLCAs during the implementation process. The HLCAs reasoned that although the online format might have increased the participation rate due to its low-threshold character, it is advantageous for communication and health literacy training sessions to take place in person.

A second crucial contextual factor that was mentioned in the interviews affecting active engagement were staff shortage and time constraints, which are a known, but nevertheless huge problem in healthcare [37–39]. This supports findings from previous studies showing that lack of resources and time are key barriers to the implementation of organizational health literacy interventions [18, 19]. Therefore, this circumstance should be considered when implementing a communication and health literacy concept that requires staff to partake in training sessions. Moreover, some of the HLCAs felt that they were facing a lack of support from leadership. This supports findings of previous literature showing that leadership support is a critical factor for organization-wide implementation of health literacy interventions [16, 18]. In this implementation effort, this could be because leaders of departments partly did not realize the extent of resources that were needed to successfully change professional health literacy, as suggested in some of the focus groups. The significance of the engagement and dedication of leaders and senior management in a healthcare organization when implementing change has previously been shown [40, 41].

A general difference in reach between the occupational groups was observed – with nurses being the most represented group in training, the survey, and the interviews. Physicians were more involved in the planning stages and only participated to a very limited extent in the later implementation and utilization of the intervention. A lack of multidisciplinary representation was mentioned in the focus groups by the hospital management and administration staff, who also pointed out that nurses seemed to be most involved in the project. This aspect was somewhat addressed by creating a training session on team communication; however, future implementation should consider increasing the degree of multidisciplinary representation. Furthermore, physicians emphasized in the interviews that they did not feel a need for communication trainings due to their work experience. However, evidence shows that years of work experience have no influence on professional health literacy, but that preparation during education and organizational conditions do [42]. This should therefore be considered in the future planning and implementation of measures to promote professional health literacy.

Every third participant stated in their survey that they found the project to be quite or fully present in the hospital, while around every fifth did not feel reached at all, which supports findings from previous implementation studies on communication interventions [43]. The focus groups showed that some of the supporting measures are unusable without the training and go unnoticed by most healthcare professionals. Furthermore, interviewees supposed that those with the highest need for communication training did not attend the training. This has implications for the voluntary nature of the training. In several interviews, the desire to make the training mandatory was expressed. This is also mentioned by Grol et al. [40] who state that exclusively voluntary actions seldom lead to success when implementing new interventions in healthcare. However, during the implementation process, the HLCAs rejected a mandatory nature of the training due to an excessive number of other duties and required instructions. Taylor et al. [43] furthermore suggest integrating the training into residents’ learning curricula rather than offering the training outside residency education to improve participation rates. A modification of this approach might be to integrate small parts of the training into routine staff meetings to reach more healthcare professionals. This was also discussed during the implementation phase, but could not be implemented within the study period because of missing flexibility in staff work routines and insufficient commitment by decision-making bodies.

The training was especially appreciated for giving a space to reflect on one’s own behavior. The contents of the training itself were often familiar to the participants, but it was the first time most of them reflected on their own professional health literacy and communication strategies. However, it might be worthwhile to evaluate the training for different professions. Some physicians believed they did not need communication training or were skeptical towards the training. Similar barriers have been observed in other studies [43–45]. The focus groups made clear that knowledge of communication techniques and the existence of supporting measures alone does not lead to utilization. Organizational framework conditions were required that support the implementation of what has been learned in everyday practice. While the training itself was evaluated positively, the translation from theory into practice was difficult, which represents a common problem already observed in other studies regarding educational programs in healthcare [46, 47]. Participants of the present study reported difficulties in remembering to appropriately use the techniques and learnings from the training and supporting measures in everyday care. After the supporting measures and communication techniques were introduced, they were barely used in routine care processes. This suggests that to target professional health literacy, even more guidance in real-life care settings is needed to sustainably change behavior such as professional communication. The wish for supervision, involving the observation of interactions between healthcare professionals and patients, was expressed by healthcare professionals to integrate the measures and learning contents into existing work processes and routines. Participants of the focus groups also suggested general changes for the future, such as reflecting on their communication during shift handovers. Unfortunately, the implementation of supervision was not feasible within the project period. Future projects could therefore integrate supervision as a feature for communication interventions targeting professional health literacy.

Despite the barriers to implementation, the participatory approach, feedback loops and co-design of the complex intervention fostered the use of the implemented measures in line with previous research [27]. This is true for the training sessions as well as the supporting measures. The participatory approach was not mentioned explicitly as a facilitating factor in the focus group interviews. However, throughout the whole project, it was observed that the strong involvement of nurses in all steps of the project might explain the higher reach of the complex intervention among nurses (e.g., higher participation rates in training sessions compared to other professions). The participatory approach showed that each hospital and even each professional group or department had different needs, and tailoring the complex intervention to those needs was important. This was most noticeable regarding the door signs, whose size worked well for one group but not for the other. In this context, the participatory approach of the study was especially beneficial to proactively adapt the measures to the individual needs of the different wards and professions.

### Implications for practice and research

Changing an organization is a difficult task that takes immense resources and time since organizations are complex systems with their own logic structures [21]. Without opinion leaders supporting interventions and acting as multipliers, change will rarely happen. Moreover, the results show how many external influences a hospital is exposed to, many of which cannot be controlled such as the Covid-19 pandemic, staff shortage, and resource constraints and a hospital merger. This process evaluation provides important insights on how to implement a communication and health literacy concept at a hospital. Future implementation of organization-wide health literacy interventions should therefore focus on tailoring the intervention even more to local challenges, on making sure that change agents from every profession and organizational level are involved, as well as on assuring leadership support from the very start. In addition to this, opinion leaders need to carry the idea of the project beyond the proposed duration and ensure that the intervention is sustained in the long term. Subsequent studies should focus on investigating the capacities of healthcare organizations for organization-wide improvement processes and help to identify how healthcare organizations can be innovative and patient-centered even in extremely difficult contextual conditions.

### Strengths, limitations and further suggestions

This process evaluation used a mixed-methods approach and was guided by a well-tested framework. Qualitative data was collected from multiple professions to enable contrasting perspectives and was used to inform the development of the quantitative survey. Both data sources were integrated in the analysis. While it was planned to recruit participants through leaders of the clinical departments, the research team, research assistants from the participating departments, and involved HLCAs, the sample process was challenging to realize mainly due to the COVID-19 pandemic and the regarding safety measures. This led to low participation rates.

At the same time, low response rates in the survey and difficulties in recruiting participants from all professions for the focus groups represent major limitations relating to validity and reliability. The low willingness to participate made it nearly impossible to conduct purposive sampling for the focus group interviews. This is also partly due to the COVID-19 pandemic, which started just before training sessions were scheduled to begin and lasted throughout the whole project, resulting in staff shortage, a high turnover of staff as well as restrictions and delays regarding intervention development, implementation and the accompanying evaluation. Thus, the complex intervention should be further tested and evaluated in the same context but also in other countries and cultures.

Besides the pandemic, another unforeseeable development was communicated by the hospital during implementation: a merger with another nearby hospital was planned and not expected. Although participants did not openly express their concerns during data collection, it was discussed in the training sessions and the steering board meetings as an event that promoted feelings of insecurity and affected priority-setting. Furthermore, the survey might have been more conclusive if more staff that participated in the training had answered the survey. Thus, measures should be taken to accomplish higher participation rates in future studies.

A further limitation of this process evaluation is that the patient perspective was not considered which should be included in future studies. Although the project followed basic principles of organizational development by including HLCAs in the change process, the study originally aimed to include patients’ perceived needs as well as barriers and wishes regarding patient communication in the process of tailoring the intervention.

In general, the reach of the complex intervention as well as the use of the intervention components appeared very limited. This could possibly be due to the circumstances under which hospitals operate, such as the pandemic, staff shortage, or resource constraints, which limit capacities and resources for innovative strength and organization-wide change. However, planned steps could not be conducted due to the COVID-19 pandemic. More flexible adaptation processes should be considered such as online training and on-the-job learning. Further formative process and summative evaluations should follow up and shed more light on the mechanisms of impact of the complex intervention.

## Conclusion

To answer the research questions, we can conclude:

1. From the point of view of the healthcare professionals, the reach of the complex intervention was rather superficial and lacked integration into organizational routines.
2. Factors promoting the implementation of the complex intervention were the participatory approach as well as HLCAs promoting the project. Factors hindering implementation were contextual factors (such as the COVID-19 pandemic, resource constraints, hospital merger), lack of support from leaders and lack of participation.
3. The healthcare professionals evaluated individual components of the complex intervention positively, but participation in the complex intervention was generally low.

The study described the implementation and process evaluation of a complex intervention aimed at improving professional health. It revealed the importance of leadership support and that change agents alone cannot create sustainable change in an organization. The process evaluation also showed that providing resources and training sessions does not necessarily change behavior.

When implementing an organization-wide intervention, it is essential to consider available resources, leadership support and change agents as well as to be responsive to the needs of different target groups. Moreover, it must be ensured that the parts of the implemented intervention are integrated in and aligned with existing work processes and routines.

## Supplementary Information

Supplementary Material 1.

Supplementary Material 2.

Supplementary Material 3.

## Data Availability

The datasets used and/or analyzed during the current study are available from the corresponding author on reasonable request.

## Abbreviations

HLCA: Health literacy change agents [German; Gesundheitskompetenzpartner:innen]
MRC: Medical Research Council
PIKoG: As made for us – Improving professional health literacy in hospitals [German; Wie für uns gemacht - Partizipativ angelegte Implementierung eines Kommunikationskonzepts zur Verbesserung der professionellen Gesundheitskompetenz]
SD: Standard Deviation
