# Supplementary material for: Process evaluation of a co-design and implementation study to improve professional health literacy in a regional care hospital (PIKoG): a mixed-methods study
Source: BMC Health Serv Res. 2025 Apr 15;25:555. doi: 10.1186/s12913-025-12679-9 (PMC12001380; doi:10.1186/s12913-025-12679-9)
Supplement: Supplementary file 1 — Supplementary Material 1. [file 12913_2025_12679_MOESM1_ESM.pdf]

Content of training session 1: Communication Skills for Patient Interaction

Topic	Aim	Time (minutes)	Content
Welcome	Creating productive, open atmosphere	35	Introductions and icebreaker
The concept "health literacy"	Introducing the topic of health literacy and demonstrate its relevance<br>Clarifying the role of the health professionals' communication skills in this process	45	• What is health literacy?<br>• Relevance of health literacy<br>• Result of low health literacy<br>• Vulnerable populations<br>• Definition of successful communication/miscommunication<br>• Importance of communication for health literacy<br>• Aims of communication<br>• Translation into practice
Health literacy in everyday practice	Answering 2 key questions:<br>• How to recognize patients' low health literacy?<br>• How to adapt communication accordingly?	35	Practical focus on health literacy and how to recognize and respond to it in everyday life:<br>• Warning signs: low health literacy<br>• Health literacy in everyday practice<br>• How can communication be patient-centered?<br>• Patient-centeredness<br>• Flexible communication<br>• Increasing flexibility
Verbal communication techniques	Developing ideas on how to prepare a good conversation<br>Understand what patient's needs are<br>Strengthening patient empowerment by using effective communication techniques	30	Focus on specific techniques/ tools:<br>• How can a good patient consultation be prepared?<br>• What do patients need?<br>• Use simple language<br>• Use additional aids (e.g., visual aids)<br>• Chunk and check/ Teach-Back<br>• Review communication techniques
Interactive communication in patient consultations	Mastering interpersonal skills (e.g. empathy, listening, honesty) in the context of patient-centered communication	80	Addressing non-verbal communication<br>• Effective communication beyond verbal communication<br>• Empathy - What is it? How can we show empathy?<br>• Can we be empathetic even when wearing a mask?<br>• Active listening skills

			• Asking open questions<br>• Paraphrasing patients<br>Interpersonal skills when delivering bad news or diagnoses<br>• Breaking Bad News<br>• SPIKES model
Non-verbal communication	Recognizing that non-verbal signals make up the largest part of communication<br>Reflecting how others perceive health professionals as communicators to promote relationships with patients	50	Examining non-verbal communication in more detail<br>• Modulating voices on the telephone<br>• How does oneself come across in conversations? How to use that?
Close	Summary and action planning	30	Revision of all topics<br>Action plan<br>Evaluation

Content of training session 2: Patient-Centered Communication

Topic	Aim	Time (minutes)	Content
Welcome	Creating productive, open atmosphere	35	Introductions and icebreaker
Health literacy and health behavior	Introduction to the topic of health literacy and its connection to health-promoting behavior and the role of healthcare professionals	40	Health literacy<br>• Types of health literacy<br>• Influences on health literacy<br>• Risk groups for low health literacy<br>Links to communication<br>• Patient-centered communication<br>• Improvement of health behavior
Diversity in the healthcare context	Consideration of patient diversity as the basis for patient-centered communication	40	• Forms and challenges of diversity in healthcare<br>• Narrative medicine<br>• Illness perceptions
Health promotion	Answering the key question: How can patients be motivated to change their lifestyle?	30	How can patients be motivated to change their lifestyle?<br>• Lifestyle change for patients with chronic diseases<br>• The path to healthy behavior<br>Strategies to support self-management<br>• Motivational interviewing<br>• The 5-A strategy
Dealing with a lack of adherence	Answering the key question: Why do many patients fail to follow the recommendations of healthcare professionals?	55	Why do many patients not follow the recommendations of healthcare professionals?<br>• (Non) compliance with recommendations<br>• Which method is the 'right' one in such a situation?<br>• Possible barriers for patients<br>• How to support patients<br>• Empowering patients and their relatives<br>What influence do patients' physical and mental impairments have on adherence?<br>• Cognitive abilities<br>• Psychological stress
Competencies in	Answering the key question: How can you be sure	45	• Caring for aggressive patients

patient-centered communication	that patients have heard and understood your instructions?		• How can we be sure that the patient has heard and understood instructions?<br>• Characteristics of attentive listening
Close	Summary and action planning	30	Revision of all topics<br>Action plan<br>Evaluation

Content of training session 3: Team Communication

Topic	Aim	Time (minutes)	Content
Welcome	Creating productive, open atmosphere	35	Introductions and icebreaker
Professional health literacy	Introducing the topic of professional health literacy and demonstrating its relevance<br>Clarifying own role in promoting professional health literacy	40	• Addressees of professional health literacy<br>• Competences of health professionals<br>• Aim of professional health literacy<br>• Relationships between professional health literacy and patients' health literacy<br>• Strengthening health literacy together
Personal role as communicator in a team	Develop a feeling for own communication<br>Self-reflection on one's own role and responsibility in communication	55	Conflicts in the team<br>Interpersonal communication characteristics<br>• Drama triangle<br>• Learning from each other<br>• Attribution theories<br>• Transactional Analysis
Diversity in the team	Emphasizing the advantages and challenges of diversity in the team<br>Realizing that there are different perceptions and that these can be explained using psychological models<br>Promoting understanding and appreciation of the other person(s)	60	• Why does diversity matter?<br>• Challenges and benefits of diversity<br>• Reference group, in-group and out-group<br>• Ethnocentrism<br>• Empathy Maps
Communication techniques in the team	Creating the conditions for a good atmosphere in the team<br>Developing own attitudes through conscious, authentic communication appropriate to the situation	55	Video example "Just a routine operation"<br>'Typical' communication problems<br>• Hierarchy and speaking-up<br>• Misunderstandings and closing-the-loop<br>• Overfocus on the task and '10 for 10'
Effective communication under time constraints	Acquiring strategies for effective and confident communication, even under time pressure and stress	30	• Structured communication and handovers<br>• The (I)SBAR concept
Close	Summary and action planning	30	Revision of all topics

Action plan<br>Evaluation
