# Supplementary material for: Process evaluation of a co-design and implementation study to improve professional health literacy in a regional care hospital (PIKoG): a mixed-methods study
Source: BMC Health Serv Res. 2025 Apr 15;25:555. doi: 10.1186/s12913-025-12679-9 (PMC12001380; doi:10.1186/s12913-025-12679-9)
Supplement: Supplementary file 2 — Supplementary Material 2. [file 12913_2025_12679_MOESM2_ESM.pdf]

Appendix 2: Supporting measures

Posters

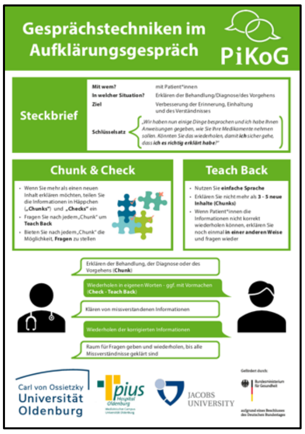

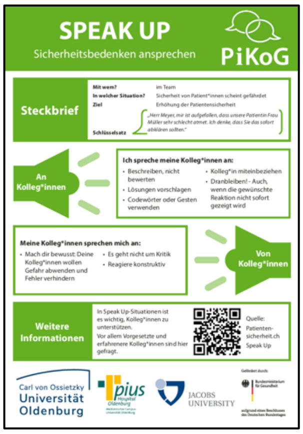

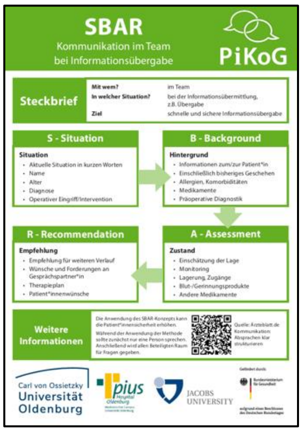

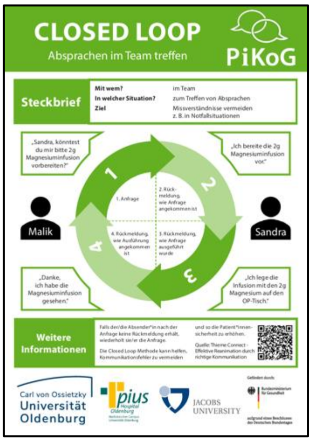

Index cards

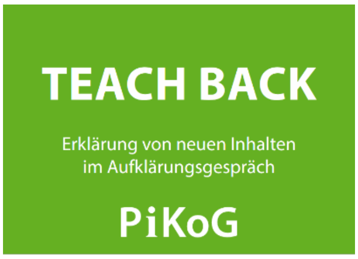

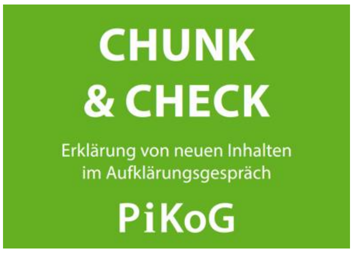

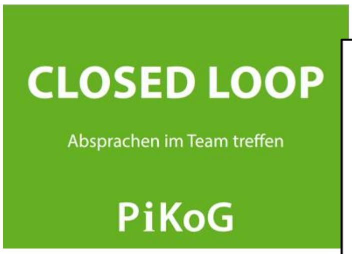

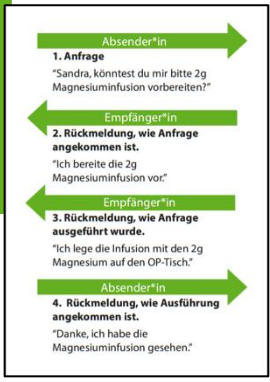

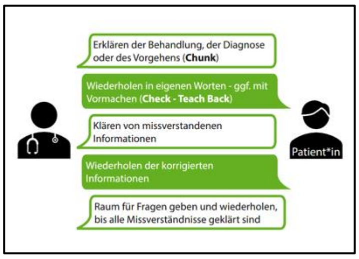

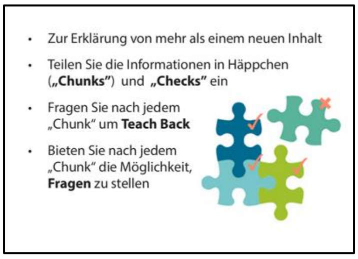

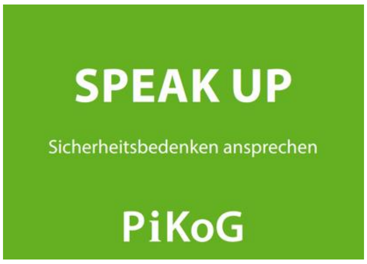

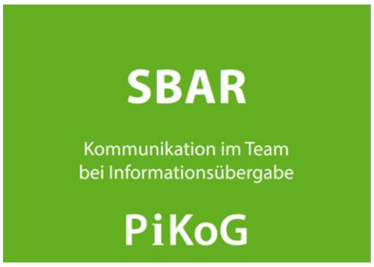

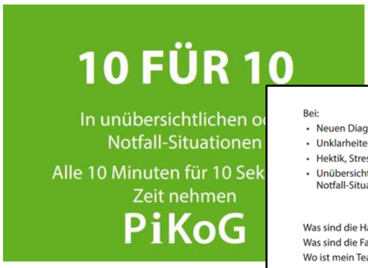

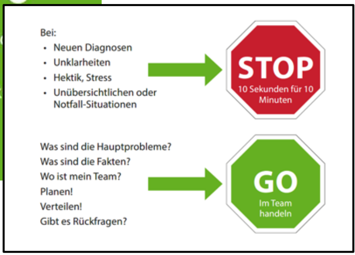

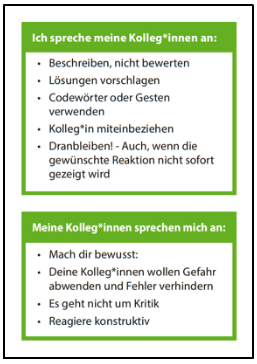

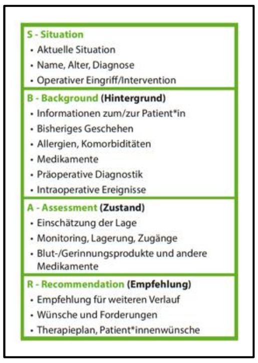

Door signs

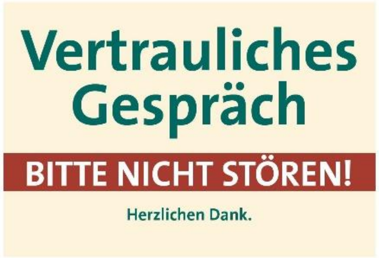

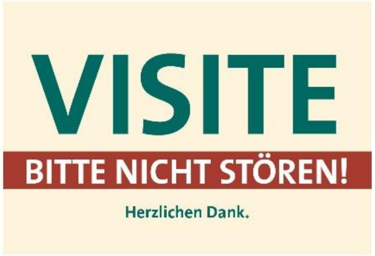

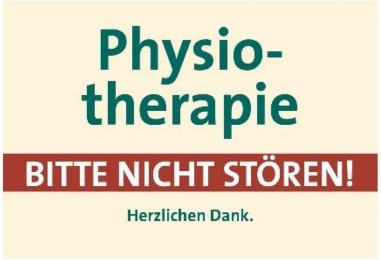

Communication cards

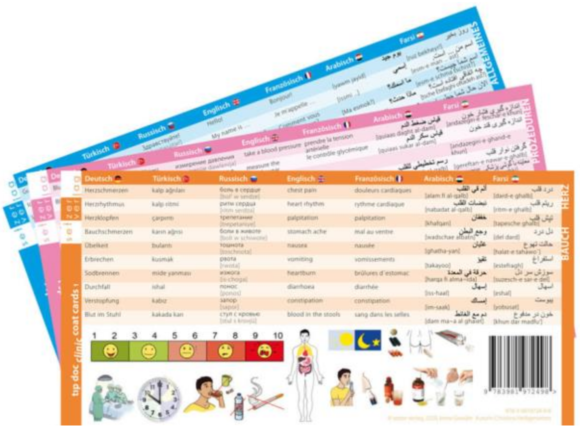

©setzer verlag e.K. http://www.setzer-verlag.com/epages/79584208.sf/de_DE/?ObjectPath=/Shops/79584208/Products/978-3-9819724-9-8

Communications portfolio

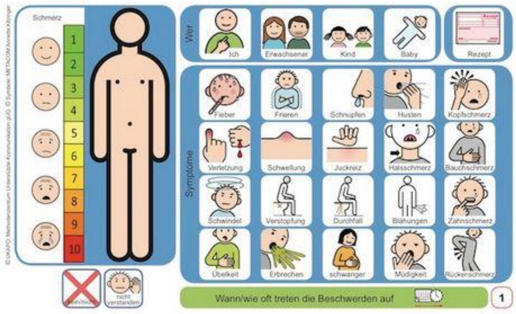

©MEZUK (Methodenzentrum Unterstützte Kommunikation)

Flyer for patients

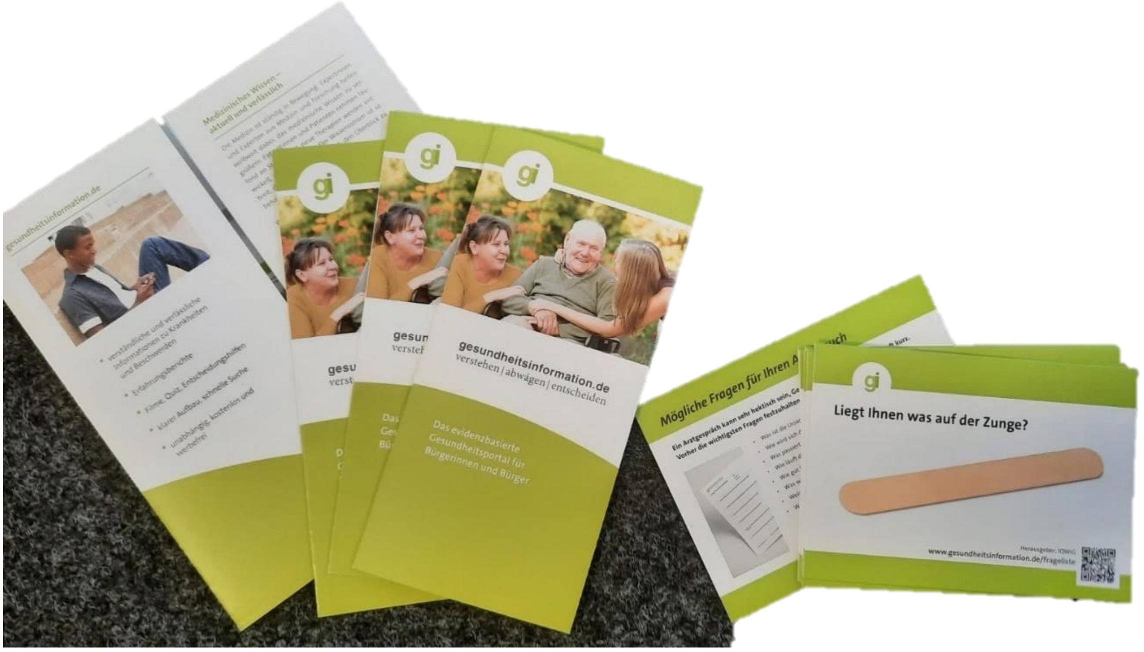

Explanations videos

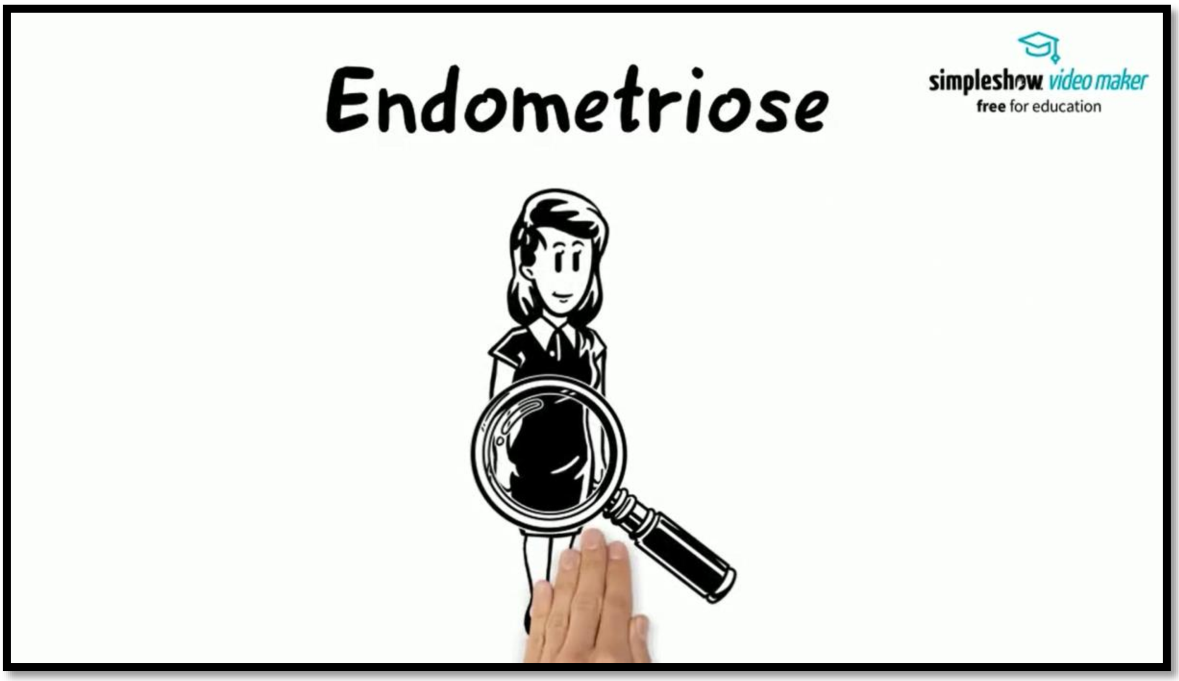

https://www.pius-hospital.de/kliniken/gynaekologie-gynaekologische-onkologie/patienteninformationen
