# Supplementary material for: Process evaluation of a co-design and implementation study to improve professional health literacy in a regional care hospital (PIKoG): a mixed-methods study
Source: BMC Health Serv Res. 2025 Apr 15;25:555. doi: 10.1186/s12913-025-12679-9 (PMC12001380; doi:10.1186/s12913-025-12679-9)
Supplement: Supplementary file 3 — Supplementary Material 3. [file 12913_2025_12679_MOESM3_ESM.pdf]

PIKoG

Interview guidelines for focus groups interviews for process evaluation

(during implementation of the communication concept)

General information

Aim	Gathering insights into the **benefits** and **feasibility** of the communication concept.<br>- Information on the implementation of the communication concept from the perspective of Pius Hospital employees<br>- Information on the status quo and possible areas for improvement
Duration of the interviews	60 to max. 90 minutes
Location	Online

Part 1: Introduction, project presentation, preparation

Duration: 5 minutes

Introduction of study team and participants, project introduction, recording device & data protection

Part 2: Focus group interview

Topic according to Moore et al. 2015	Key question	In-depth questions	Indicators
**Description of the intervention**<br><br>**Implementation**<br><br>**Duration:**<br><br>20 minutes	- What have you noticed about the PIKoG project so far?<br><br>We sent you all the accompanying measures that we have implemented on the wards. In addition, communication trainings on the following 3 topics has been offered over the past 9 months: communication skills for patient interaction, patient-centred communication, team communication.<br><br>- Which of the supporting measures do you know/have you already seen on the ward and, if applicable, already used?<br><br>- Have you participated in one or more of the trainings?<br><br>- How did integrate the supporting measures and the learning content of the trainings in you daily clinical routine?<br><br>- What did/would it have taken to participate in the trainings/use the supporting measures?	- How did you get to know about it?<br><br>- If no measures known/used: what could be the reason? If measures are known: How do you rate the use of the flanking measures?<br><br>- If no: What could be the reason for this? If yes: How would you rate the trainings?<br><br>- Ask about all accompanying measures:<br><br>- Were there any changes at the hospital that were not part of the communication concept that had an influence on participation in training/the use of accompa-	What was delivered: fidelity/dose<br><br>Reach/dose<br><br>How was the intervention implemented?<br><br>Context

		nying measures?	
**Mechanisms of impact**<br><br>**Outcome**<br><br>**Duration:**<br><br>20 minutes<br><br>(start after 10 minutes with interactive part)	- Has the implementation of the communication concept changed anything for you?<br>- Has the implementation of the communication concept changed anything in the hospital?<br>- How do you rate the benefits of the communication concept, especially with regard to the project's goals of improving the health literacy of patients and the hospital as a whole?<br><br>We now have a small, more interactive task for you on this topic.<br>Last year, before the start of the project, we carried out surveys in which we asked, among other things, what wishes you had for **improved communication with patients**. As you can see, we have now written these wishes on cards. We will now go through each wish with you and ask you to decide whether the desired aspect has either deteriorated, not changed at all or improved as a result	- Changes in the communication with the patients<br>- Changes regarding the conditions of conversations with patients (e.g. fewer interruptions)<br>- Changes in team communication<br>- Changes in the use of supporting measures to improve/facilitate communication<br>- Reactions of patients (e.g. enquiries during conversations with patients)<br>- In your opinion, which measures of the communication concept (training and/or one or more of the supporting measures) have brought the greatest benefit for communication with patients?	Interaction with intervention<br><br>Change of outcomes

	of the communication concept		
**Adaptions**<br><br>**Duration:**<br><br>15 minutes	- Do you see a need to change the training/supporting measures?<br><br>- Do you have any suggestions for improving or adapting the training/supporting measures?	- To what extent might the training content need to be improved?<br><br>- To what extent might organizational aspects of the training need to be improved?<br><br>- To what extent might the supporting measures need to be improved??	Need for adaptions
**Closure**	We have now reached the end of our focus group interview.<br>With this in mind, I would like to thank you once again for your participation today and for the lively discussion! In doing so, you have helped us to identify the benefits of and need for adaptation to the communication concept.<br><br>If you have any further questions, please feel free to ask them, or if you have any questions afterwards, please do not hesitate to contact us at any time.		

References

Moore GF, Audrey S, Barker M, Bond L, Bonell C, Hardeman W, et al. Process evaluation of complex interventions: Medical Research Council guidance. BMJ. 2015;350:h1258. doi:10.1136/bmj.h1258.
